# Mediators in Internet-Based Cognitive Behavior Therapy for Severe Health Anxiety

**DOI:** 10.1371/journal.pone.0077752

**Published:** 2013-10-17

**Authors:** Erik Hedman, Erik Andersson, Gerhard Andersson, Nils Lindefors, Mats Lekander, Christian Rück, Brjánn Ljótsson

**Affiliations:** 1 Department of Clinical Neuroscience, Division of Psychiatry, Karolinska Institutet, Stockholm, Sweden; 2 Department of Clinical Neuroscience, Osher Center for Integrative Medicine, Karolinska Institutet, Stockholm, Sweden; 3 Department of Clinical Neuroscience, Division of Psychology, Karolinska Institutet, Stockholm, Sweden; 4 Department of Behavioural Sciences and Learning, Swedish Institute for Disability Research, Linköping University, Linköping, Sweden; 5 Stress Research Institute, Stockholm University, Stockholm, Sweden; McGill University, Canada

## Abstract

According to the cognitive behavioral model of severe health anxiety (hypochondriasis) four central maintaining mechanisms are how the individual perceives the risk of disease and how negative its consequences would be, attention to bodily sensations, and intolerance of uncertainty. The aim of the present study was to investigate the mediating role of these putative mechanisms in Internet-delivered CBT for severe health anxiety. We analyzed data from an RCT where participants were randomized to Internet-delivered CBT (n=40) or to a control condition (n=41). Mediators and outcome, i.e. health anxiety, were assessed weekly throughout the treatment, enabling fulfillment of the criterion of temporal precedence of changes occurring in the mediator in relation to the outcome to be met. The results showed that reduced perceived risk of disease, less attention to bodily symptoms, and reduced intolerance of uncertainty significantly mediated improvement in health anxiety. The study supports the validity of the cognitive behavioral model of health anxiety. The findings have theoretical and clinical implications as they indicate processes that may be causally related to the improvements observed after CBT for health anxiety.

## Introduction

Severe health anxiety is highly prevalent in health care settings, associated with long-term disability, and an increased risk of developing major depression, and often chronic if untreated [1,2,3,4]. In the present paper severe health anxiety is defined as hypochondriasis according to DSM-IV stipulating that the disorder is marked by a persistent fear of developing serious somatic disease [5]. This fear stems from misinterpretation of normal bodily sensations. Cognitive behavior therapy (CBT) has been shown to be an effective treatment for severe health anxiety and has substantial empirical support [6,7,8,9,10]. In a randomized controlled trial, our research group showed that CBT delivered via the Internet can produce large and enduring effects in the treatment of severe health anxiety [11,12]. Internet-based CBT could be described as clinician-guided online bibliotherapy and is based on the idea that it is possible to achieve the same behavioral changes via Internet-delivered treatment interventions as in conventional CBT [13]. This is achieved through extensive self-help texts, called modules, combined with structured assignments that could be tailored to the individual’s needs. Thus, the main principle is that participants in Internet-based CBT are exposed to the same treatment components as in conventional CBT. An Internet-based secure platform is used to publish modules, to provide possibility for online communication between participant and therapist, and to store work sheets and online assessments. 

According to the cognitive behavioral model, there are several putative factors involved in the maintenance of health anxiety and thus potentially important to affect in psychological treatment of the disorder. First, a central proposed mechanism is the stable tendency among persons with severe health anxiety to misinterpret bodily sensations and other ambiguous health stimuli as signs of serious disease [14]. Results from a recent empirical study indicates that persons with health anxiety are more likely to perceive bodily sensations as indicators of catastrophic illness, when compared to individuals with other anxiety disorders and healthy controls [15]. A second important suggested cognitive process is how the individual perceives the consequences of the disease [14]. That is, even if one believes that a certain bodily sensation has a low probability of indicating HIV a perception that the consequences of the disease would be unbearable could lead to high levels of health anxiety. A third maintenance mechanism of the cognitive behavioral model is that increased attention to bodily sensations contributes to higher detection rates of sensations caused by normal harmless processes, which would otherwise have passed by unnoticed [16]. This increased internal focus leads to exposure to a larger amount of stimuli that could be misinterpreted as evidence of threat. Intriguingly, evidence indicates that this form of attentional bias could be further strengthened by attempts to divert the attention from bodily reactions, suggesting that it could be part of a hypervigilance-avoidance response pattern [17]. It has also been shown that, among patients with non-cardiac chest pain, persons with anxiety disorders are significantly more vigilant to and fearful of cardiac sensations compared to non-anxious controls [18]. The fourth putative mechanism is intolerance of uncertainty, which has been shown to be associated with health anxiety and with outcome in CBT for generalized anxiety disorder [19,20]. According to Buhr and Dugas [21], intolerance of uncertainty may be defined as an excessive inability to accept that a negative outcome may occur, regardless of how small the probability of the event is. 

Although these factors have been suggested to play a central role in the maintenance of health anxiety, it is unclear whether CBT achieves its effects by altering these processes, i.e. if they mediate the effect of treatment. A mediator is an event that occurs after treatment onset but precedes outcome, is associated with the independent variable, and has a main or interactive effect on treatment outcome [22,23]. Investigating mediators of treatment effect is important to understand both psychopathological and therapeutic mechanisms and may ultimately lead to development of more effective treatments [23]. In light of the above, perceived risk and negative consequences of disease, attention to bodily sensations and intolerance of uncertainty could all be viewed as plausible mediators of effect in Internet-based CBT for severe health anxiety. Other mechanisms, besides the four described, suggested by the cognitive behavioral model are avoidance and safety behaviors, but they were not investigated in the present study.

To our knowledge, no previous study has prospectively investigated whether the four putative CBT mechanisms outlined above mediate treatment effect in conventional or Internet-based CBT for severe health anxiety. More knowledge in this area could contribute to an increased understanding of treatment mechanisms.

 The aim of the present study was to investigate mediators of treatment effect in Internet-based CBT for severe health anxiety using data from a previously reported clinical trial where participants were randomized to either Internet-based CBT (n=40) or to a control condition that did not receive active treatment (n=41) [12]. We hypothesized that reductions in perceived risk and negative consequences of disease, lessened attention to bodily sensations, and increased tolerance of uncertainty would mediate the effect of treatment on treatment outcome. Specifically we predicted that:

- There would be a direct effect, i.e. the treatment would lead to larger reductions in health anxiety compared to the control condition,
- There would be an effect of treatment on the mediators (a-path), i.e. in comparison to the control condition, the treatment would lead to superior reductions in perceived risk and negative consequences of disease, lessened attention to bodily sensations, and increased intolerance of uncertainty,
- There would be an effect of the mediators on health anxiety (b-path),
- The direct effect of treatment on health anxiety would be reduced after controlling for the mediators.

## Methods

### Design

 This study employed a randomized controlled design where participants were allocated to Internet-based CBT (n=40) or to a control condition on waiting list with access to a discussion forum (n=41) in a 1:1 ratio. Thus, there was experimental control of the effect of treatment condition (Internet-based CBT vs. control condition) on the outcome and mediators. The main findings of the RCT has been previously published and the trial was registered at ClinicalTrials.gov, ID NCT00828152 [12]. Participants spent the same amount of time, 12 weeks, in their respective condition. Assessments of both mediators and outcome were conducted on a weekly basis throughout the 12-week treatment period, which meant that an appropriate time line could be established. The trial was approved by the regional ethics review board in Stockholm and conducted in accordance with the guidelines of the Declaration of Helsinki. Participants provided written informed consent electronically over the Internet.

### Main inclusion criteria and recruitment

The study was conducted at a clinic specialized on Internet-based CBT [24], which is located in a university hospital in Stockholm, Sweden. Participants were recruited through self-referral and through referral from primary or psychiatric care. To be included in the study, participants had to (a) have a primary diagnosis of severe health anxiety, i.e. hypochondriasis, according to DSM-IV [5], (b) agree not to undergo any other psychological treatment for the duration of the study, (c) have a constant dosage two months prior to treatment if on prescribed medication for anxiety or depression and agree to keep dosage constant throughout the study, and (d) have no history of psychosis or bipolar disorder. A detailed description of the recruitment process and the participants is provided in the main outcome study [12].

### Characteristics of the Participants

 A description of the participants is presented in Table 1. 

**Table 1:** Description of the participants.

**Variable**		**Internet-based CBT participants**	**Control condition**
		**(n=40)**	**(n=41)**
**Age**	Mean age (SD)	39.3 (9.8)	38.8 (9.5)
**Gender**	Women (%)	28 (70%)	32 (78%)
	Men (%)	12 (30%)	9 (22%)
**Severe Health Anxiety**	Mean duration in years (SD)	20.0 (13.8)	22.0 (12.4)
**Comorbid psychiatric disorder at baseline**	Any anxiety disorder (%)	20 (50%)	21 (51%)
	Depression or dysthymia	6 (15%)	8 (20%)

Abbreviations: CBT, Cognitive behavior therapyAssessment of outcome and mediatorsThroughout the treatment, the short version of the Health Anxiety Inventory (SHAI) [25] was administered weekly. The SHAI consists of the 18 items most highly correlated with the full scale and has excellent psychometric properties including high internal consistency (Cronbach’s α=.95), good test-retest reliability over three weeks (*r*=.87), and high convergent validity with other health anxiety measures [25,26,27]. The respondent is asked to rate the items based on his or her experience over the past week. In the present study, this was considered a sound rationale for administering the scale on a weekly basis.

To assess mediators while ensuring that the change in the mediator occurred before the change in the outcome, we used an approach splitting the SHAI into one health anxiety outcome scale and four mediator scales. All subscales were based on the scale dimensions suggested by the originators of the SHAI based on a priori factors that were identified as important taking into account the phenomenology of severe health anxiety as well as cognitive behavioral theory [25]. As the SHAI is designed to assess core features of the cognitive behavioral model of severe health anxiety, i.e. suggested maintenance mechanisms or putative mediators, it was regarded as the best available instrument for mediator assessment. The outcome scale comprised the total score of the two subscales “Disease conviction” and “Fear and worry about illness”. These scales were chosen as outcome as these dimensions both from a conceptual and diagnostic perspective (DSM-IV) strongly reflect the core features of severe health anxiety. Also, in the widely used health anxiety measure Whiteley Index, the same type of scales, i.e. “Illness conviction” and “Illness worrying” constitute two thirds of the instrument thus indicating that these constructs are the most central dimensions of health anxiety [28]. The four mediator subscales each corresponded to a potential mediator. Perceived risk and negative consequences of disease were assessed with the subscales “Perceived vulnerability to illness” and “Negative consequences”, respectively. Attention to bodily sensations was assessed with the subscale “Bodily awareness” and intolerance of uncertainty was measured by the subscale “Psychological reactions to bodily sensations”. As the latter scale is not an established measure of intolerance of uncertainty the findings regarding this factor should be viewed as tentative. The scale is comprised of SHAI items 10 and 13 where the extreme response alternative of the former item is “If I have a bodily sensation or change I must know what it means” and the corresponding response alternative of item 13 is “If I notice an unexplained bodily sensation I always find it difficult to think of other things”. 

### Diagnostic assessment

To assess whether participants met diagnostic criteria for severe health anxiety and other axis-1-disorders we used the Health Anxiety Interview [16] and the Mini International Neuropsychiatric Interview (MINI) [29]. 

### Internet-based CBT

The treatment consisted of a self-help text of 118 pages provided in 12 weekly modules through an Internet-based treatment platform with access to a therapist via a secure online contact system. Participants were granted access to subsequent modules by their therapist. Each module was devoted to a specific theme and included homework exercises. The treatment platform was used as a mean of providing the tools and knowledge necessary for conducting *in vivo* behavior change viewed as important from a CBT-perspective. 

The treatment was based on a cognitive behavioral model for health anxiety, emphasizing the role of avoidance and safety behaviors, internal focus, and interpretations of bodily sensations as signs of serious illness as maintaining factors of health anxiety [16,30]. The main component of the treatment was exposure and response prevention. The treatment also included mindfulness training as a means of acquiring the skill to experience bodily sensations without trying to control them or seek reassurance. This was done to facilitate exposure and to lessen attempts to divert attention from aversive bodily sensations, a potential form of safety behavior that may counteract exposure [17,31]. Particular benefits of mindfulness training in cognitive therapy for severe health anxiety have been shown recently [32,33]. Of note, mindfulness in the present study was not used as a stand-alone intervention but was used specifically to optimize effectiveness of exposure and response prevention exercises. In brief, modules 1 and 2 comprised psychoeducation about CBT and health anxiety and introduced mindfulness training, module 3 focused on cognitive processes in health anxiety, the main component of modules 4-10 was exposure to health anxiety stimuli and response prevention, and modules 11 to 12 were focused on maintaining treatment gains and relapse prevention. The treatment protocol was developed by our research group and it has been validated in a trial investigating the effects of group CBT for severe health anxiety [34]. 

 Patient and therapist had no face-to-face contact during the treatment. The therapists conducting internet-based CBT were four licensed psychologists to whom participants were randomly assigned. All therapists received supervision by the first author (E.H.) throughout the trial. 

### Statistical Analysis

Statistical analyses were conducted using SPSS version 20.0 (SPSS inc., Chicago). Mediation was tested in four steps within a mixed effects models framework using criteria for mediation as suggested by Baron and Kenny [22] meaning that the there must be: (I) a direct effect of treatment (Internet-based CBT vs. control condition) on the outcome (health anxiety), (II) a significant effect of treatment condition on the mediator, (III) a significant effect of the mediator on the outcome, and (IV) a reduction of the impact of the direct effect (Internet-based CBT vs. control condition) on the outcome when controlling for the mediators. In these analyses, time, treatment group and mediator were modeled as fixed effects, but did not include random slope or intercept. The randomized design of the study made it possible to have experimental control of the effect of Internet-based CBT on the mediator, which has been suggested to be an important criterion for mediation by Smits et al. [35]. In order to assess the prerequisite of temporal precedence of the mediator, e.g. [36], the outcome at weeks 2 to 12 was entered as the dependent variable and the potential mediators (i.e. perceived risk and negative consequences of disease, lessened attention to bodily sensations, and intolerance of uncertainty) as lagged time-varying predictor variables (T-1), i.e. assessed at weeks 1 to 11. To control for the effect of the outcome score at the previous week (T-1) when estimating the predictive effect of the putative mediator on the outcome at time T all models contained the outcome scores from weeks 1 to 11 as covariates. Analyses of missing data were conducted using Little's Missing-Completely-At-Random test (MCAR) [37]. Effect sizes were calculated using Cohen’s *d* based on pooled standard deviations. 

## Results

### Attrition and Adherence

There was no data loss at pre-treatment or post-treatment. During the treatment phase 765 of 891 (86%) assessments were completed. According to MCAR, data was missing completely at random (χ^2^_(230)_ = 217.1, *p*=.72). On average, participants receiving Internet-based CBT completed 9.1 (SD=3.3) modules. 

### Summary of Efficacy of Internet-based CBT

**Figure 1:**
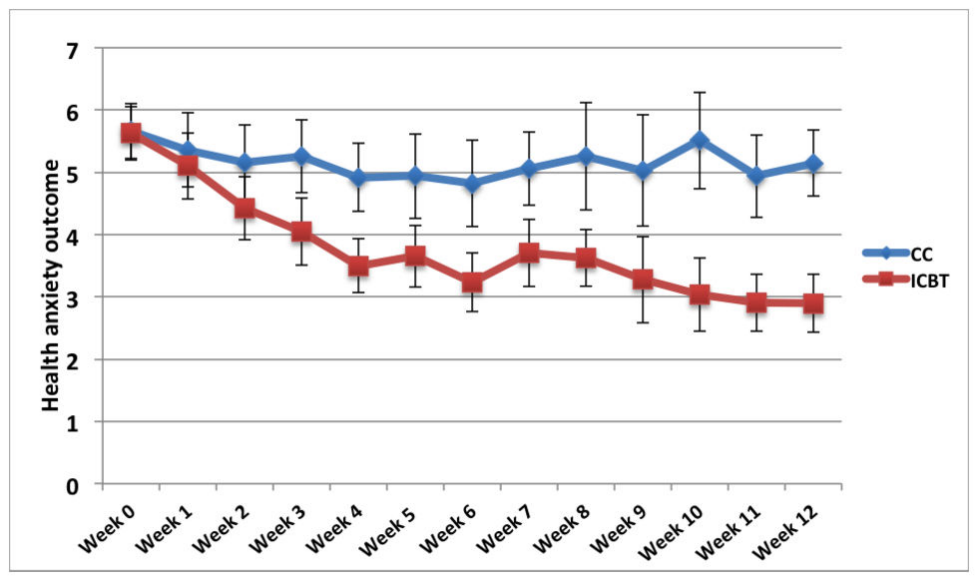
Weekly course of improvement throughout the treatment period. Abbreviations: ICBT, Internet-based cognitive behavior therapy; CC, control condition. Note 1: Error bars represent 95% conficence intervals. Note 2: the outcome measure is comprised of the Disease conviction scale and Fear and worry about illness scale of the Short-Health Anxiety Inventory.

Between-group effect sizes were large on the primary outcome measure HAI (full scale) at post-treatment (d= 1.62; 95%CI=1.10-2.10). Effects were also large when using the aggregated score of the “Disease conviction” and “Fear and worry about illness” subscales of the SHAI, which was the outcome in the mediational analyses in this study (between-group d=1.38; 95%CI=0.89-1.85). Figure 1 displays the weekly course of improvement in health anxiety during the treatment period. As reported in the main outcome study, participants receiving Internet-based CBT also made moderate to large improvements on secondary measures of depressive symptom and general anxiety. 

### Mediational analyses

Table 2 displays means and SDs of the four putative mediators and the outcome across the assessment period. Intercorrelations among mediators and outcome at baseline are presented in Table 3.

**Table 2:** Means and SDs on measures of health anxiety and mediators as assessed by the Short Health Anxiety Inventory.

		**Outcome**		**Mediators**							
		**Health anxiety**		**Perceived risk of disease**		**Perveiced awfulness of disease**		**Attention to bodily sensations**		**Intolerance of uncertainty**	
		Scale range (0-9)		Scale range (0-3)		Scale range (0-12)		Scale range (0-6)		Scale range (0-6)	
**Time point**	**Group**	**M**	**SD**	**M**	**SD**	**M**	**SD**	**M**	**SD**	**M**	**SD**
**Week 1**	ICBT	5.10	1.68	2.10	0.55	5.20	2.38	4.05	0.92	3.97	1.22
	CC	5.36	1.82	2.06	0.63	6.25	2.67	4.22	1.17	3.86	1.13
**Week 2**	ICBT	4.42	1.59	2.03	0.55	5.24	2.17	3.87	0.96	3.42	1.43
	CC	5.16	1.74	2.06	0.56	5.91	2.56	4.28	0.81	4.13	0.94
**Week 3**	ICBT	4.05	1.76	1.81	0.46	4.59	2.50	3.86	1.06	3.30	1.41
	CC	5.26	1.78	2.03	0.71	6.09	2.31	4.11	1.23	3.97	1.36
**Week 4**	ICBT	3.50	1.37	1.71	0.52	4.29	2.51	3.53	1.03	2.97	1.46
	CC	4.92	1.71	1.89	0.57	5.86	2.42	4.11	1.00	3.86	1.18
**Week 5**	ICBT	3.66	1.56	1.76	0.54	4.32	2.41	3.45	1.03	2.89	1.39
	CC	4.94	1.98	2.06	0.66	6.09	2.10	4.21	1.17	3.85	1.52
**Week 6**	ICBT	3.24	1.48	1.63	0.59	4.11	2.59	3.37	1.05	2.45	1.33
	CC	4.82	2.08	2.00	0.55	6.15	2.39	4.03	1.17	3.68	1.22
**Week 7**	ICBT	3.71	1.61	1.65	0.49	4.03	2.46	3.32	0.98	2.74	1.31
	CC	5.06	1.77	2.11	0.58	6.37	2.65	4.09	1.10	3.97	1.15
**Week 8**	ICBT	3.63	1.44	1.55	0.56	4.03	2.45	3.42	0.95	2.63	1.22
	CC	5.26	2.28	2.07	0.55	5.70	2.33	4.19	1.15	4.07	0.87
**Week 9**	ICBT	3.28	2.00	1.34	0.75	3.38	2.67	3.22	1.36	2.59	1.72
	CC	5.03	2.50	2.07	0.79	6.00	2.96	4.13	1.25	4.10	1.35
**Week 10**	ICBT	3.03	1.70	1.41	0.71	3.59	2.70	3.06	1.19	2.31	1.40
	CC	5.51	2.34	2.03	0.79	6.06	2.95	4.03	1.32	4.06	1.33
**Week 11**	ICBT	2.91	1.36	1.36	0.74	3.48	2.51	2.73	1.04	2.21	1.52
	CC	4.94	1.97	1.85	0.61	5.79	2.40	3.94	1.21	3.79	1.12
**Week 12**	ICBT	2.90	1.50	1.35	0.66	3.40	2.57	2.85	1.03	1.95	1.26
	CC	5.15	1.74	1.98	0.61	5.95	2.52	4.02	1.08	3.66	1.02

Abbreviations: ICBT, Internet-based cognitive behavior therapy; CC, control condition.

**Table 3:** Intercorrelations of the outcome and the mediators at baseline.

**Measure**	**Correlations** (Pearson, zero-order)				
	**1.**	**2.**	**3.**	**.4**	**5.**
**1. Health anxiety**	-				
**2. Perceived risk of disease**	.49	-			
**3. Perceived awfulness of disease**	.25	.35	-		
**4. Attention to bodily sensations**	.36	.39	-.01	-	
**5. Intolerance of uncertainty**	.23	.10	.16	.32	-

#### Effect of the treatment on the outcome (direct effect)

Mixed effects model analyses showed that there was a significant direct effect, i.e. interaction effect of treatment and time on the outcome (*F*_(12, 112)_=3.5, *p* < 001), indicating superior improvements in the Internet-based CBT group in comparison to the control group.

#### Effect of the treatment on the mediators (a-path)

Analyses showed that there was a significant interaction effect of treatment and time the on all mediators, i.e. perceived risk of disease (Perceived vulnerability to illness scale; *F*_(12, 122)_=2.9, *p* = .002), perceived negative consequences of disease (Negative consequences scale; *F*_(12, 131)_=2.3, *p* = .01), attention to bodily sensations (Bodily awareness scale; *F*_(12, 117)_=1.9, *p* = .04), and intolerance of uncertainty (Psychological reactions to bodily sensations scale; *F*_(12, 139)_=5.0, *p* < .001).

#### Effect of the mediators on outcome (b-path)

Mixed effects model analyses showed that was a significant effect of all mediators on the outcome (F_(1, 745-869)_=77.8-701.7, *p* < .001), i.e. lower perceived risk and less negatively perceived consequences of disease, lesser attention to bodily sensations, and higher intolerance of uncertainty was associated with lower levels of health anxiety.

#### Test of mediation

When adding the putative mediators in the mixed effects models comprising treatment and time, there was a change in the direct effect, i.e. of treatment on outcome in three of four putative mediators. When including perceived risk of disease as covariate, there was no longer a significant interaction effect treatment and time on outcome (*F*_(12, 126)_=1.6, *p* = .10). The same reduction of the direct effect was found, i.e. it was no longer significant, when including attention to bodily sensations as covariate (*F*_(12, 131)_=1.8, *p* = .51) and when including intolerance of uncertainty as covariate *F*_(12, 136)_=1.5, *p* = .12). When controlling for the mediator negative consequences of disease, the direct effect of treatment on outcome however remained significant (*F*_(12, 113)_=2.2, *p* = .02). To assess the criterion of temporal precedence, mixed effects models analyses were conducted investigating the effect of each mediator at week T-1 on the outcome at week T controlling for outcome scores at week T-1. The analyses showed that the mediators perceived risk of disease, attention to bodily sensations, and intolerance of uncertainty remained significant (F_(3-10, 183-600)_=3.3-6.2, *p* = .001-.02). Perceived negative consequence of disease did not have a significant impact on outcome at week T while controlling for outcome at week T-1 (*F*_(12, 582)_=1.4, *p* = .14). We also conducted analyses to investigate whether there were bidirectional effects. The results showed that outcome at time T-1 was associated with mediators perceived risk of disease, attention to bodily sensations, and intolerance of uncertainty at time T controlling for the mediators at time T-1 (F_(9, 579-615)_=5.2-6.7, *p* < .001), suggesting that improvement in health anxiety also led to subsequent change in the mediators. There was no significant effect of outcome at time T-1 on the negative consequences of disease at time T controlling for the mediator at time T-1 (*F*_(9, 578)_=1.7, *p* = .10). Taken together, these results indicate that the treatment affected all putative mediators and that perceived risk of disease, attention to bodily sensations, and intolerance of uncertainty mediated treatment outcome, while perceived negative consequences of disease did not mediate outcome.

## Discussion

To our knowledge this study is the first to investigate the mediating role of the suggested cognitive behavioral mechanisms perceived risk and negative consequences of disease, attention to bodily sensations, and intolerance of uncertainty, in CBT for severe health anxiety. We analyzed data from an RCT comparing Internet-based CBT to a control condition and found that reduced perceived risk of disease, less attention to bodily symptoms and increased tolerance of uncertainty mediated improvement in health anxiety while reduced perceived negative consequences of disease did not mediate the effect of CBT. Important strengths of the study were the randomized design allowing for test of the causal effect of the treatment on the mediators and outcome, the well-validated outcome measures and the weekly assessment of mediators and outcome.

In general, the findings of the present study suggest that improvement in CBT for severe health anxiety is at least partially mediated by change in the maintenance mechanisms proposed by the cognitive behavioral model. In line with this model, we hypothesized that perceived risk and negative consequences of disease would be associated with subsequent change in health anxiety [38]. The results showed that although the treatment caused significant changes in these mediators, only perceived risk of illness mediated participants’ improvements in health anxiety while perceived negative consequences of disease did not. The former result matches findings in research on social anxiety disorder showing that reductions in the perceived probability of adverse social events predicted symptom severity after treatment with CBT [39,40]. However, in the area of social anxiety reduced estimated cost of the feared social outcome, which could be viewed as the analog to the concept perceived negative consequences of disease in the present study, was shown to be a mediator of the effect of CBT [41]. The results of this study also contrast with mediators in CBT for panic disorder, where major improvements are often achieved through altering negative interpretations of panic attacks, i.e. patients become less anxious after learning that an attack *per se* is not dangerous [42]. So, how is this difference to be understood? One possible explanation may be that in severe health anxiety the feared outcome is typically highly aversive in an objective sense (i.e. having a serious disease with a relatively high probability of death). This is likely to leave less room for improvement by reducing the negativity bias in interpretations of the severity of the feared outcome. In this perspective, it is less surprising that reductions in health anxiety were more related to a reduced perceived risk of disease than to perceived negative consequences. A clinical implication of these findings is that interventions would benefit from tackling perceived risk of disease rather than the consequence of the disease as an important factor for improvement. As this is probably the first study investigating these factors within a mediational framework in CBT for severe health anxiety it is however central to underscore that the findings need to be replicated. We do not suggest that the findings of this study give sufficient evidence to claim that perceived consequences of disease is not important to take into account. Notably, negativity bias in terms of perceived risk of disease was a significant mediator of treatment outcome in a treatment whose main intervention was exposure and response prevention. This suggests that components aimed directly at targeting cognitive processes, such as behavioral experiments and cognitive restructuring, are not necessary to bring about cognitive changes that are important for reduction in health anxiety. Thus, somewhat speculatively, it might be that an efficient way of reducing perceived risk of disease could be to use exposure to health anxiety stimuli while using mindfulness techniques.

The results also showed that reduced attention to bodily reactions mediated the treatment effect, i.e. reduced internal focus predicted reduction of health anxiety. These are interesting findings, as the treatment comprised a fairly large amount of mindfulness training aimed at enhancing exposure by breaking the hypervigilance-avoidance pattern. This could mean that systematic training in maintaining internal focus when distressed by bodily sensations may actually reduce attention to bodily sensations in the long run. This in turn, leads to health anxiety improvements. As the treatment in the present study entailed a large amount of exposure (interoceptive, in-vivo as well as imaginative exposure), it could also be that exposure in itself led to reductions in attention to bodily symptoms. The rationale for including mindfulness training in this study was to enhance exposure, i.e. to increase the probability that participants could maintain in contact with aversive internal stimuli while conducting exposure exercises. Thus, we regard it as possible that exposure exercises in combination with mindfulness training could interact to produce reduced attention to bodily sensations. 

As proposed by Dugas and colleagues, intolerance of uncertainty also mediated the effect of the treatment [43]. This meant that participants who showed larger reduction in their need to know the meaning of unexplained bodily sensations also displayed larger subsequent improvements in health anxiety. This suggests that it might be beneficial to take the uncertainty aspect into consideration when designing exposure and response prevention exercises. The results in this regard should however be interpreted with caution as we did not use a conventional measure of intolerance of uncertainty in the assessment of this mediator. However, we view the SHAI subscale “Psychological reactions to bodily sensations”, which was used in the present study, as a fair marker of this construct.

 In the present study, two potentially highly important components of the cognitive behavioral model, avoidance and reassurance seeking, were not investigated in the meditational analyses. Thus, we regard it as important that future studies investigate whether these behaviors have a mediating role in the treatment of CBT. Another import topic for future investigation is whether the mechanisms of CBT differ between persons with DSM-V somatic symptom disorder (SSD) and illness anxiety disorder. Considering that the DSM-IV criteria of hypochondriasis used in this study have a stronger resemblance to the SSD criteria than to illness anxiety disorder it could be that the findings of this study to a somewhat larger extent are generalizable to persons with SSD.

 This study has several strengths and limitations, which are discussed in the following and compared against the four mediation quality criteria proposed by Smits et al. [35]. These quality criteria stipulate that (a) statistical mediation should be demonstrated, (b) that the treatment should cause the effect on the mediator, (c) the mediator should cause the change in the outcome, and (d) there should be specificity in the relation between the mediator and the outcome. In the present study, the criterion for statistical mediation (a) was met as there was a direct effect of treatment on outcome, an effect of treatment on the three mediators, an effect of the mediators on outcome, and lastly, the direct effect was reduced after controlling for the mediators. The b-criterion was met as the randomized controlled design made it possible to conclude that the change in the mediators were caused by the treatment and was not due to a third confounding variable. The c-criterion was met in so far that the study used a design with weekly assessments of both mediator and outcome and that statistical analyses controlling for the outcome at time T-1 showed that mediator status at time T-1 predicted outcome at time T. It should however be noted that this was a bidirectional relationship, meaning that the outcome also mediated three of the investigated mediators. This could be indicative of a clinical upward spiral process where changes in the mediators leads to reductions in health anxiety, which in turn leads to behaviors that further affect the mediators and so forth. One other comment regarding the c-criterion is that although the temporal precedence criterion was met, it is still not possible to strictly claim causal effect of the mediator on the outcome. As pointed out by Smits et al. [35], causality of the mediator needs to be demonstrated using a design where manipulation of the mediator is done. A venue for future research could thus be to investigate the effects of directly manipulating the mediators studied in this paper. 

The present study did not meet criterion d, i.e. that there was a specificity of the mediator-outcome relationship. That would have required weekly assessment of processes hypothesized to be unrelated to outcome, which was not done in the present study. However, the fact that one of the investigated variables, perceived negative consequences of disease, did not mediate outcome suggest that there was a degree of specificity concerning the other three mediators. Another limitation of the present study, unrelated to the Smits et al. criteria, was that as the assessments were conducted on a weekly basis this meant that for a mediator to be identified using the temporal precedence criterion, there had to be a one week delay of effect after change in the mediator. The design would have failed to discover shorter mediator-outcome intervals, i.e. if change in health anxiety occurred within a few days or hours after change in the mediator. However, as our hypotheses were largely supported by the data, this is probably less of a problem in the present study. Finally, the limitations of the measures used should be mentioned. Although the SHAI has demonstrated good psychometric qualities it should be acknowledged that the mediator scales used in this study was a priori constructed by the originators of the SHAI and not developed through factor analysis. This meant that there is an increased risk of intercorrelations among the mediators affecting the outcome, and the fact that the negative consequence scale of the SHAI has been shown to be a distinct construct [27] while being a non-significant mediator variable in the present study could partly suggest that the findings was affected by limited eigenvalues of the mediators. Regarding assessment of mediators it should also be mentioned that self-report measures in the assessment of body vigilance has been shown to exhibit psychometric limitations [e.g. 44]. 

 Despite the limitations described above we view the findings of this study to be important as they show, for the first time, that perceived risk of disease, attention to bodily sensations, and intolerance of uncertainty are mediators of treatment effect in Internet-based CBT for severe health anxiety. These findings support the cognitive behavioral model of health anxiety and are of value to the clinician as they identify key processes in achieving improvement in the treatment of severe health anxiety. 
